# A Novel Metal-Containing Mesoporous Silica Composite for the Decolorization of Rhodamine B: Effect of Metal Content on Structure and Performance

**DOI:** 10.3390/nano12234108

**Published:** 2022-11-22

**Authors:** Yasaman Ghaffari, Md Saifuddin, Suho Kim, Soyoung Beak, Jiyeol Bae, Kwang Soo Kim

**Affiliations:** 1 Department of Environmental Research, Korea Institute of Civil Engineering and Building Technology (KICT), University of Science and Technology (UST), Daejeon 34113, Republic of Korea; 2 Department of Environmental Research, Korea Institute of Civil Engineering and Building Technology (KICT), Goyang 10223, Republic of Korea; 3 Civil and Environmental Engineering Department, Hanyang University, Seoul 04763, Republic of Korea; 4 Department of Civil & Environmental Engineering, Yonsei University, Seoul 03722, Republic of Korea

**Keywords:** Fenton-like reaction, silica-based catalyst, organic pollutants, photocatalysis, neutral pH

## Abstract

A series of novel Mn_x_Fe_y_@SiO_2_ (x,y = 1–20%) nanocomposites were synthesized for the first time via the sol-gel/combustion method with different content of precursors (Mn and Fe acetate salts). The effect of precursor content and ratio on physicochemical properties were observed by various characterization methods. Moreover, Rhodamine B (RhB) was chosen as the target pollutant to test the performance of these nanocomposites under a photocatalytic Fenton-like reaction. The results showed that the nanocomposite morphology improved by increasing Fe and Mn content. In this study, interesting behavior was observed in BET results which were different from the fact that increasing metal content can decrease the surface area. This study revealed that one metal could be more critical in controlling the properties than another. Moreover, the precursor ratio appears to have a more tangible effect on the surface area than the effect of precursor content. Among all synthesized nanocomposites, Mn_1_Fe_5_@SiO_2_ showed the highest surface area of 654.95 m^2^/g. At optimum batch conditions (temp = 25 °C, catalyst dosage = 1 g L^−1^, H_2_O_2_ = 75 mmolL^−1^, and initial RhB concentration = 50 mg L^−1^), complete removal (simultaneous adsorption/degradation) occurred using Mn_1_Fe_5_@SiO_2_ at neutral pH. This study showed that the designed nanomaterial could be used as a dual functional adsorbent/photocatalyst in different environmental applications.

## 1. Introduction

Dyes are considered organic pollutants, which are known for their harmful effects on the environment and ecosystem. According to the annual worldwide dye production database, over 10,000 types (over 700,000 tones) of dyes are manufactured every year [1]. Dyes have considerable applications in several industries, including printing, pulp mill, textile, electroplating, and cosmetics, which cause severe water contamination. Most common carcinogenic products, such as benzidine, are present in organic dyes that must be removed before they discharge into wastewater and other water sources [2]. Nowadays, the removal of organic pollutants like dyes has become a concern for environmental scientists. Various treatment techniques have been established, such as coagulation or flocculation, ultra-filtration, reverse osmosis, and biodegradation. However, most of these conventional approaches have faced several drawbacks. Therefore, it is essential to find new methods to remove organic pollutants [3]. Rhodamine B(RhB) is an amphoteric dye employed in food and fabric dyeing industries and belongs to the xanthene dyes class. RhB is one of the most challenging organic pollutants to degrade and control. According to IUPAC nomenclature, Rhodamine B is called N-[9-(ortho-carboxyphenyl)-6-(diethylamino)-3H-xanthen-3-yli-dene] diethyl ammonium chloride and has a high molecular weight of 479.02 g mol^−1^ [4].

Catalytic oxidation/reduction is one of the most useful methods to degrade organic dyes where catalysts such as biocatalysis, COF (covalent organic framework), MOF(metal-organic framework), TiO_2_, carbon nanotubes, zeolites, etc., have been reported in the literature [5,6,7]. However, due to their complicated synthesis, low thermal stability, high energy consumption, and expensive precursors, applying them on a real scale is an unpractical approach. Furthermore, most photocatalysts can be utilized only in the acidic pH, which requires pre and post-treatment to fulfill environmental regulations. Gao Y. et al., 2017 prepared MIL-53(Fe) by solvothermal method and used it for the removal of clofibric acid and carbamazepine from water. MIL-53(Fe)/H_2_O_2_/vis exhibited high photocatalytic activity at pH 3, and the photocatalytic activity decreased noticeably at neutral pH [8]. Farrokhi A. et al., 2019 synthesized Phosphonate-based MOF materials, namely STA-12 (Fe), and tested them for the degradation of methylene blue (MB) and methyl Orange (MO) in the aqueous solution. The effect of solution pH on the removal efficiency of dyes over STA-12 (Fe)/sunlight/H_2_O_2_ system was examined at 30 °C. The efficiency was highest in the acidic condition, and the degradation decreased significantly with the increase of pH, and, at pH 10, the reaction stopped [9]. 

In this regard, nanocomposites have gained extensive attention due to widespread usage in different fields, such as water and wastewater treatment, energy storage devices, the pharmaceutical industry, solar cell manufacturers, semiconductor industries, and hydrogen production [10]. Nanocomposites are multiphase solid materials improving performance in a wide range of pH. For this purpose, heterostructure nanocomposites like polymer-metal matrix nanocomposites, carbon-supported metal oxide nanocomposites, metal matrix nanocomposites, and modified metal matrix nanocomposites have been reported. CuO–ZnO [11], NiO–Al_2_O_3_ [12], FeNi_3_/SiO_2_/CuS [13], C/NiO–ZnO [14], Chitosan-graphene oxide iron oxide [15], PANi/Bi_2_WO_6_ [16], metal oxide loaded on activated carbon [17], and Rectorite-supported Fe_3_O_4_ composite [18] are examples of these nanocomposites. 

Li. X. et al., 2017 fabricated g-C_3_N_4_/NH_2_-MIL-88B (Fe) heterojunction for visible light-induced Fenton-like excitation of H_2_O_2_ for methylene blue (MB) degradation. The results showed that this nanocomposite could successfully degrade MB after 120 min in pH7 [19]. Arghavan F. et al., 2021 prepared nickel ferrite/chitosan/bismuth(III) oxyiodide nanocomposite for metronidazole degradation. The results showed that metronidazole was completely removed at neutral pH and a reaction time of 200 min [20].

Among all the nanocomposites, SiO_2_ based nanomaterials have gained much interest due to the abundance of precursors, simple and fast synthesis methods, and high porosity. Additionally, SiO_2_ has the advantage of good thermal stability, which makes it an ideal support material [21]. Having a high surface area can give adsorbent characteristics to the nanomaterial. However, adding metal compounds to the structure is necessary to have catalytic properties. Solution Combustion Synthesis (SCS) is a redox-based reaction that occurs in an aqueous environment in the presence of oxidizing (e.g., metal nitrates, acetates, carbonates) and reducing agents (organic fuels such as hydrazine, EDTA, glycine). The reaction occurs through the oxidation of fuels to pore-making gases such as CO_2_, N_2_, and H_2_O. The liberated gases can crack large particles and form porous nanomaterials, further improving the active phase distribution and the catalyst structural and thermal stability. Fuels can connect with metal ions to start stable chelate complex compounds, which allows for attaining a more homogeneous mixture and avoids segregations, which is very advantageous for designing multi-component oxides [22].

Considering the above advantages and disadvantages, this study reported a simple and economical synthesis of heterostructure Mn_x_Fe_y_@SiO_2_ (x,y = 1–20%) nanocomposite using the sol-gel/combustion methodology. To the best of our knowledge, this nanomaterial has never been reported before. Detail physicochemical characterization techniques such as field emission scanning electron microscopy (FE-SEM), High-resolution transmission electron microscope (HR-TEM), Thermogravimetric analysis (TGA), N_2_ adsorption-desorption isotherm, X-ray diffractometer (XRD), Fourier-transform infrared (FTIR), Ultraviolet-visible light-diffuse reflectance spectroscopy (UV–Vis DRS), X-ray photoelectron spectroscopic (XPS), and electron spin resonance (ESR) were carried out. The application of this material was tested by the degradation of Rhodamine B (RhB) under a photocatalytic Fenton-like reaction. The influence of various experimental parameters such as catalyst dosage, initial concentration of RhB, UV power, and H_2_O_2_ concentration on the degradation process was also investigated.

## 2. Materials and Methods

### 2.1. Chemicals and Reagents

Manganese(II) acetate (>98%), Iron(II) acetate(98%), Cetyltrimethylammonium chloride (CTAC) solution (25 wt.%), and Rhodamine B (RhB) were supplied by Sigma Aldrich, Darmstadt, Germany. Glycine was procured by Yakuri Pure Chemicals Co. Ltd., Kyoto, Japan. Hydrogen peroxide (28% H_2_O_2_) solution was procured from Daejung Chemicals and Metals Co. Ltd., Siheung, Korea. Tetraethyl Orthosilicate 95% (TEOS) was purchased from the Samchun Company, Seoul, South Korea. All the chemicals were used without any further purification.

### 2.2. Synthesis of Mn_x_Fe_y_S photocatalysts

A series of novel Mn_x_Fe_y_@SiO_2_ (x,y = 1–20%) nanocomposites were synthesized for the first time via the sol-gel/combustion method with different content of precursors (Mn and Fe salts). The Mn_x_Fe_y_@SiO_2_ nanocomposites were fabricated according to our previously published method with modifications in precursors and conditions [23,24]. Iron-Manganese containing amorphous silica (Mn_x_Fe_y_@SiO_2_) was synthesized using the sol-gel/combustion technique at room temperature. Different percentage of Manganese(II) acetate and Iron(II) acetate was dissolved in 60 mL of CTAC solution and mixed for 15 min. After gaining a homogenous solution, 1 g of glycine was added to the mixture and stirred for 30 min. Next, 60 mL of TEOS solution was added to the solution and mixed for another 30 min. The solution was transferred to the incubator and stirred at 25 °C and 200 rpm for 24 h to form a gel. The gels were dried at 70 °C for 4 h in an oven. Finally, Mn_x_Fe_y_@SiO_2_ was obtained by calcination at 550 °C for 6 h under air. It should be noted that depending on the precursor content, the gel showed different colors (Table 1). 

### 2.3. Instruments

All the instruments used in this study are mentioned in Supplementary Data.

### 2.4. Experimental Procedure

The utilized photoreactor in this experiment consisted of one quartz tube in the middle and four UV-C lamps around it. The UV lamps were low-pressure mercury lamps with a power of 8 W and an intensity of 0.5 W/m^2^. A variable voltage transformer was used to adjust the UV light intensity of the UV lamps. In order to prevent the effect of heat in the removal experiment, the solution temperature was kept constant (25 °C) using cooling fans installed at the bottom of the photoreactor. It should be noted that it was designed in black to minimize the influence of other light sources in the experiments. The prepared nanocomposites were examined for their ability to degrade RhB under photocatalytic Fenton-like reactions. In a typical experiment, 1g L^−1^ of the nanocomposites was added to a 200 mL RhB solution with (50 mg L^–1^) concentration. Then, 75 mmol L^–1^ of 28% H_2_O_2_ was poured into the previous solution. At desired time intervals, 4 mL of the RhB solution was taken to analyze the removal performance using UV-Vis spectroscopy (LAMBDA 365 UV/Vis Spectrophotometer, Perkin Elmer).

## 3. Results and Discussion

### 3.1. Physicochemical Characteristic of Nanocomposites

**Figure 1:**
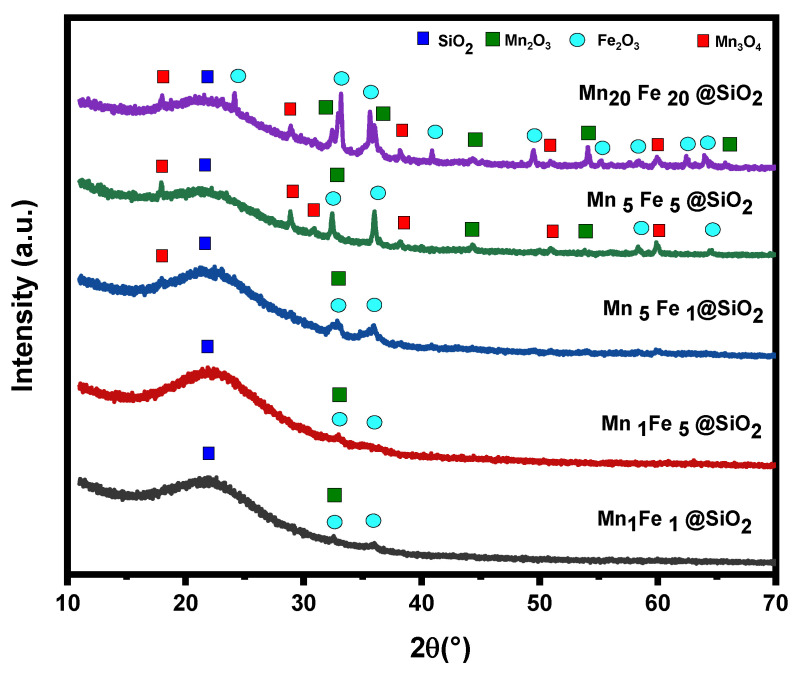
XRD patterns of M_x_F_y_@SiO_2_ nanocomposites.

**XRD**: The composition of the Mn_x_Fe_y_@SiO_2_ nanocomposites was identified using the XRD technique (Figure 1). The indicated peaks can be indexed to Mn_2_O_3_-Fe_2_O_3_ @ SiO_2_. A broad diffraction peak at 2θ of 21.63° represents amorphous silica [25]. The nanocomposites with lower Fe and Mn content (Mn_1_Fe_1_@SiO_2_, Mn_1_Fe_5_@SiO_2_, and Mn_5_Fe_1_@SiO_2_) showed low-intensity peaks with a smaller number of peaks; while, for the samples with higher content of metal (Mn_5_Fe_5_@SiO_2_ and Mn_20_Fe_20_@SiO_2_), peaks were visible with higher intensities. Nine diffraction peaks at 2θ = 24.15°, 33.16°, 35.67°, 40.92°, 49.42°, 54.29°, 57.40°, 62.52°, and 64.0° were assigned to the structure of *α*-Fe_2_O_3_ (JCPDS No. 33-0664) with their miller indices plane of 012, 104, 110, 113, 024, 116, 018, 214, and 300, reflections, respectively. Five diffraction peaks at 2θ = 32.33°, 36.1º, 44.38º, 54.04º, and 65.78º were assigned to the structure of *α*-Mn_2_O_3_ (JCPDS No. 24–508) with their miller indices plane of 222, 400, 332, 440, and 622 reflections, respectively [26]. Furthermore, in the sample with higher Mn content, Mn_3_O_4_ was also observed [27]. 

**Textural properties:** The configuration and microstructure of the Mn_x_Fe_y_@SiO_2_ nanocomposites were analyzed by field emission scanning electron microscopy (FE-SEM) and high-resolution transmission electron microscope (HR-TEM) images (Supplementary Figures S1 and S2). Spherical-like particles were observed in all the images, and by the increasing Fe and Mn concentrations, the spherical structure improved and became more uniform (Supplementary Figure S1). The HR-TEM image of nanocomposites has been illustrated in Supplementary Figure S2. The Fe_2_O_3_/Mn_2_O_3_ nanoparticles could be observed in the silica. 

**Figure 2:**
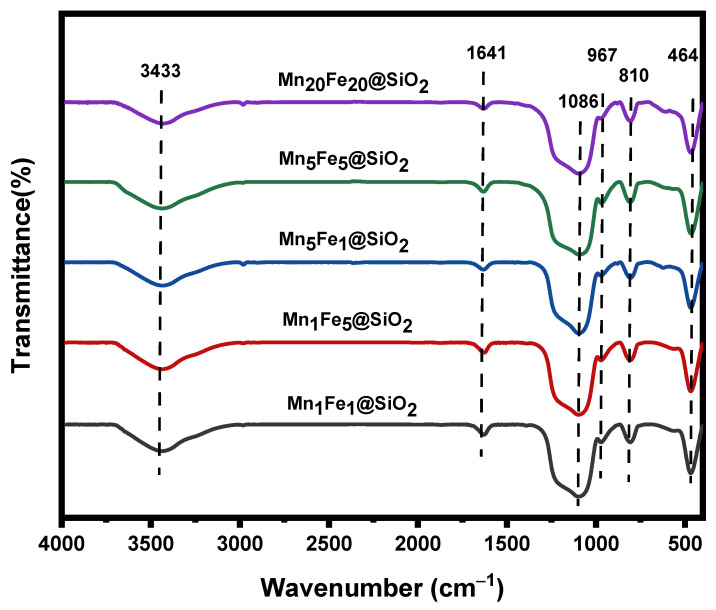
FTIR spectra of the synthesized nanocomposites.

**FTIR**: The FTIR analysis was carried out to identify the functional groups on the surface of Mn_x_Fe_y_@SiO_2_ nanocomposites (Figure 2). Peaks at 464 cm^–1^, 810 cm^–1^, 967 cm^−1^, 1086 cm^–1^, 1641 cm^–1^, and 3433 cm^–1^ are observed in the transmission spectra of all nanocomposites. The absorption band at 1086 cm^–1^ was related to the antisymmetric stretching of Si–O–Si. The symmetric mode of Si–O–Si appeared at 810 cm^–1^. Moreover, the stretching vibration of Si–OH (967 cm^–1^) and bending of Si–O (464 cm^–1^) are also observed. The peak centered at 967 cm^–1^ confirms the presence of silanol groups on the silica surface or pores in all the nanocomposites. The small peak at 1641 cm^–1^ and the broad peak at 3433 cm^–1^ are due to the stretching vibration of OH groups in absorbed physical water and Si–OH, respectively. The Si–O band always appears around 461 cm^–1^, which here is covered by stretching vibration of the metal-oxygen bond in the range of 469 cm^–1^~480 cm^–1^ [18,23]. Soubaihi R. et al., 2021 studied Pd/SiO_2_ Catalyst characteristics, and similar peaks for SiO_2_ have been reported [28].

**Figure 3:**
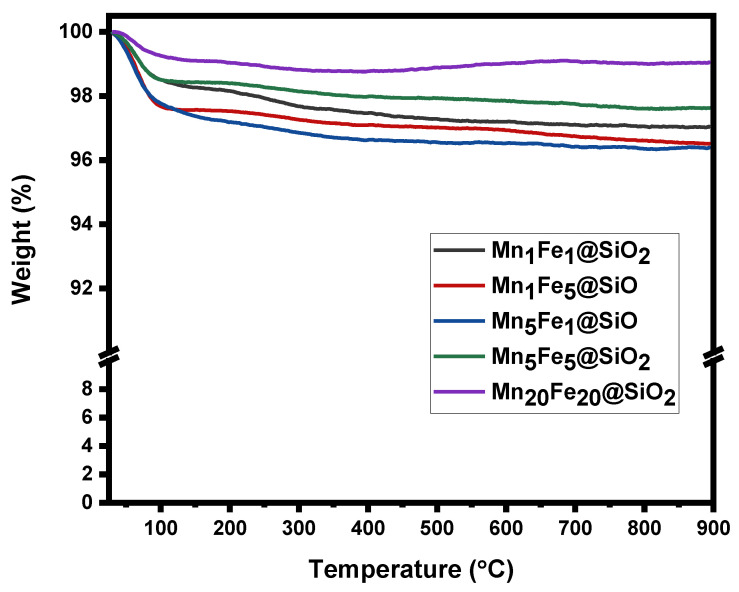
Thermogravimetric analysis TGA.

**TGA:** The thermal stability of the nanocomposites is shown in Figure 3. The samples were heated from 25 °C to 900 °C, and weight loss in the samples was reported. An insignificant weight loss was observed at <100 °C in all nanocomposites due to the adsorbed moisture on the nanoparticle surface. Moreover, the weight loss in higher temperatures was also insignificant (2–4%), showing that the prepared nanocomposites were thermally stable.

**Figure 4:**
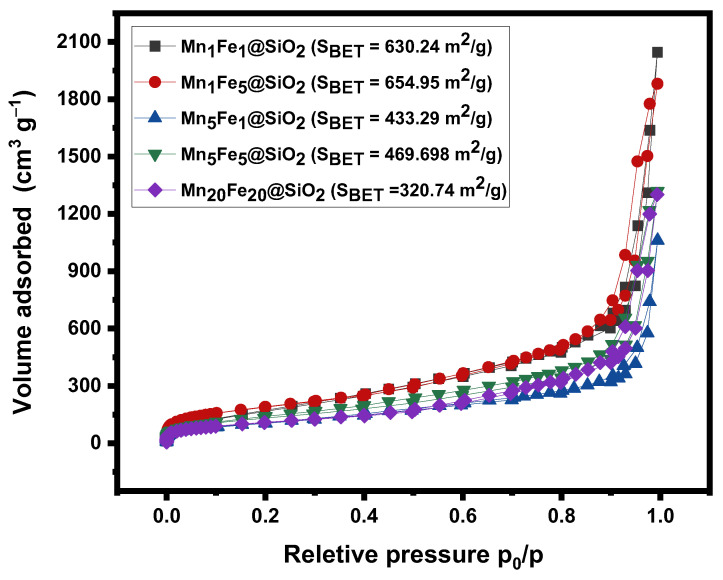
N_2_ adsorption-desorption isotherms.

**BET:** Porosity characteristics of nanocomposites were tested by N_2_ adsorption-desorption isotherm (Figure 4). Based on IUPAC classification, synthesized nanocomposites show N_2_ sorption isotherms of Langmuir type IV isotherm and are considered as a H2(b) type hysteresis loop, which verifies their mesoporous nature [29]. The specific surface areas (*S*_BET_) were calculated using the Barrett–Joyner-Halenda method. The results showed that the sample showed a surface area in the range of 320.74 m^2^/g–654.95 m^2^/g. The fact that surface area decreased with an increasing metal fraction is reported in many studies [30], and the lowest surface area was expected to be M_20_Fe_20_@SiO_2_. However, in this study, among all the nanocomposites, the lowest surface area (*S*_BET_ = 320.74 m^2^/g) belonging to the sample M_5_Fe_1_@SiO_2,_ which has a high Mn and low Fe content, and the highest specific surface area of 654.95 m^2^/g belonged to the sample with more Fe and less Mn content (M_1_Fe_5_@SiO_2_). From these results, it can be concluded that increasing Mn has a more substantial effect compared to Fe in decreasing the surface area. Moreover, the ratio of Mn and Fe is more important than their content in nanocomposite.

**Figure 5:**
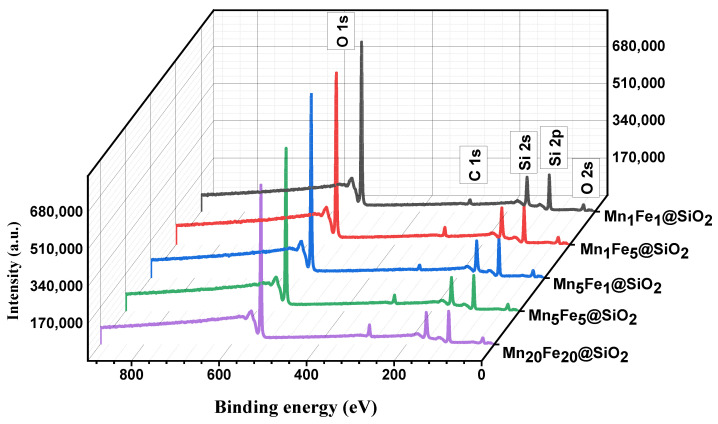
HRXPS Full spectrum of nanocomposites.

**XPS:** the oxidation state of elements in the prepared nanocomposite was analyzed using XPS. The XPS spectra showed Si2p, O1s, Mn2p, and Fe2p, which demonstrated the presence of these elements in the prepared samples. In this study, the Fe and Mn content is low, and the related peaks were not visible in full spectra in Figure 5.

**Figure 6:**
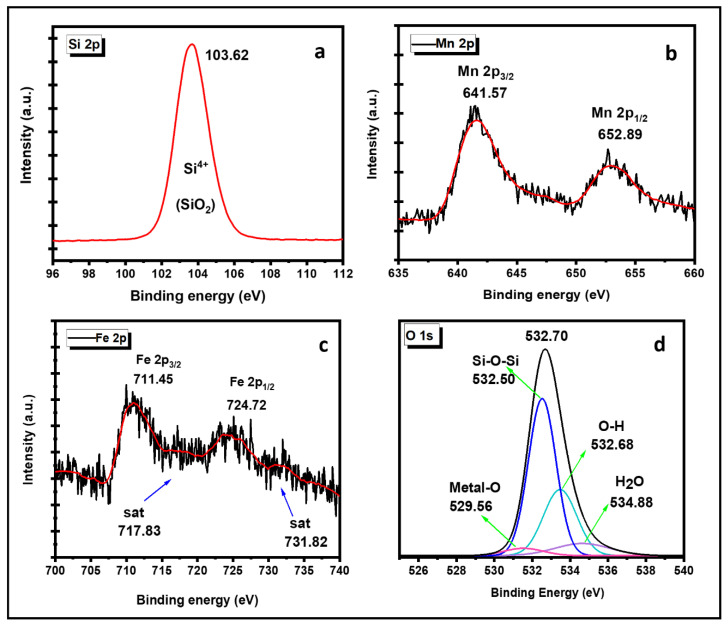
(**a**) HRXPS Si 2p spectrum; (**b**) HRXPS Mn 2p spectrum; (**c**) HRXPS Fe 2p spectrum; (**d**) HRXPS O 1s spectrum Mn_1_Fe_5_@SiO_2_.

In the Si spectrum, a single peak appeared at binding energies (B.E.) of 103.62 eV belonging to the O–Si–O bond related to the Silica network (Figure 6a). The Mn spectra showed two distinguishable peaks observed at B.E. of 641.57 eV for Mn2p_3/2_ and 652.89 eV for Mn2p_1/2_, which corresponded to the Mn (III) oxidation state (Figure 6b) [31]. Hazarika K. et al. 2018 reported that the spin energy separation of the Mn^3+^ oxidation state is 11.7 eV. Similar results have been observed in this study, and the spin energy separation of Mn^3+^ was found to be 11.3 eV [32]. Similarly, at the Fe 2p spectrum, two peaks at 711.45 eV for Fe 2p_3/2_ and 724.72 eV for Fe 2p_1/2_ were observed, which were a fingerprint of Fe(III) in the nanocomposites (Figure 6c) [33]. The shakeup peaks observed at the higher binding energy near the main peaks were assigned to the satellite peaks of Fe(III) oxidation states, which are shown by blue arrows. For the HRXPS O 1s peaks were located at B.E of 529.56 eV, 532.50 eV, 532.68 eV, and 534.88 eV, which could be attributed to the Metal–Oxygen (Metal: Fe and Mn), silica–Oxygen(Si–O), surface hydroxyl (O–H), and adsorbed H_2_O, respectively [34]. The evidence of Si–O–Si or M–O bonds formed in the composite was confirmed by the FTIR shown in Figure 2.

**Figure 7:**
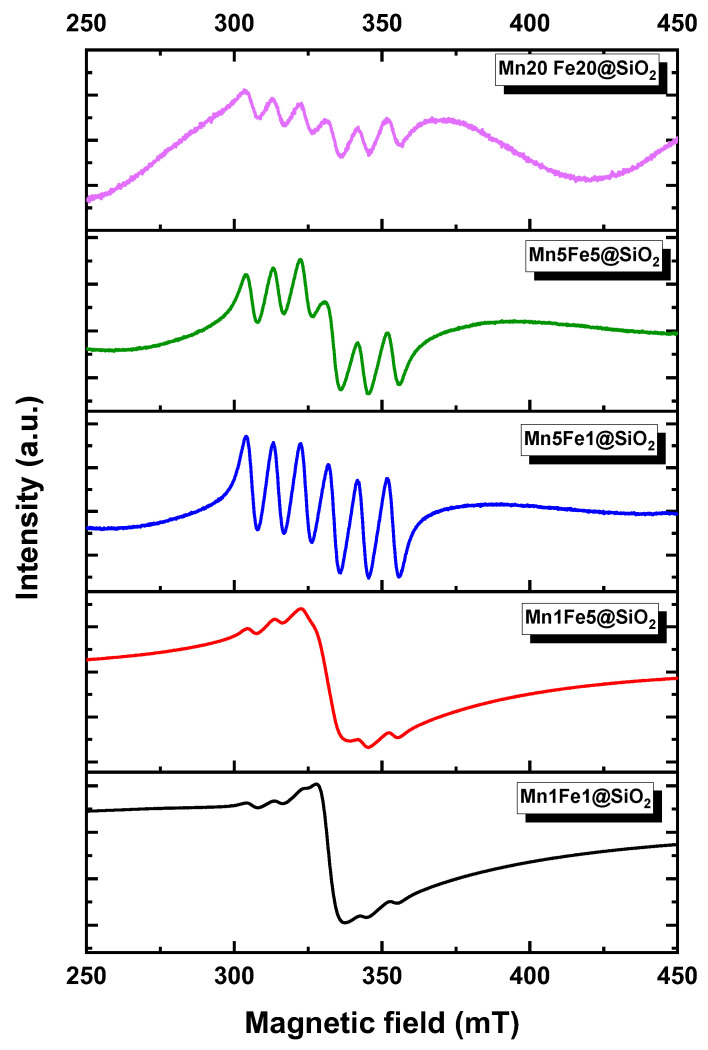
ESR spectra recorded at 298 K.

**ESR:** To further understand the elemental state of the nanocomposites, ESR analysis was conducted at room temperature (Figure 7). The high-intensity signal at 332 mT was assigned to be *g* = 1.98, which was attributed to the existence of the Fe^3+^ ions in all the nanocomposites. The sextet was observed approximately at 306 mT, 314 mT, 323 mT, 344 mT, and 354 mT, which were attributed to *g* = 2.16, 2.1, 2.03, 1.91, and 1.86, respectively. 

Based on the catalyst composition, the presence of Mn(III) (Mn_2_O_3_) was expected. However, Mn(III) is ESR silent, and the spectra do not appear as a sextet. Therefore, the observed sextet shape of spectra could be due to the presence of the Mn(II) (Mn_3_O_4_) in a mixture with Mn(III) (Mn_2_O_3_) [35,36]. These results are in accordance with the XRD result, which showed the presence of mixed Mn valence (Mn_2_O_3_ and Mn_3_O_4_) in nanocomposites with high Mn fractions. It should be noted that in the XRD spectra of the nanocomposites with low Mn concentration, the Mn_3_O_4_ was not visible. However, ESR analysis showed the structure with more detail and accuracy and proved that even nanocomposites with low Mn contents Mn_3_O_4_ exist. 

**Figure 8:**
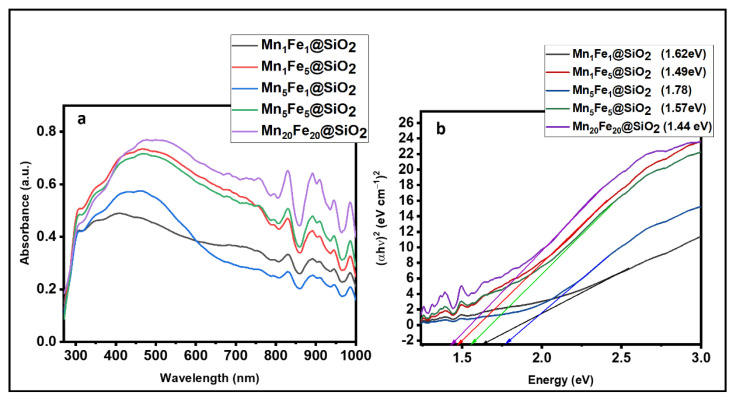
(**a**) UV-Vis DRS spectra; (**b**) bandgap energy.

**UV–Vis DRS:** The optical properties and bandgap of synthesized nanocomposites were studied using UV–Vis DRS (Figure 8a,b). The spectrum showed that all the nanocomposites could absorb the whole spectrum of UV, visible, and IR. The observed multiple peaks were related to the absorption bands of Mn_2_O_3_ and Fe_2_O_3_ in the SiO_2_.

Region 1 (250–400 nm) mainly results from the ligand to metal charge-transfer transitions, and Region 2 (400–600 nm) is considered to be the result of the pair excitation process (^6^A_1_(^6^s) + ^6^A_1_(^6^s) to ^4^T_1_(^4^G) + ^4^T_1_(^4^G)). Region (600–750 nm) is assigned to the ^6^A_1_(^6^s) to ^4^T_2_(^4^G) transition at about 650 nm, and Region 4 (750–900 nm) is the ^6^A1(^6^s) to ^4^T_1_(^4^G) transition at about 830 nm. Moreover, the band in the 375–440 nm range is attributed to the d-d transitions of Mn^3+^ in Mn_2_O_3_ [37].

The optical bandgap can be calculated using the equation αhυ = A(hv − E_g_)^n^, Where hν is photon energy, α is absorption coefficient, and n represents ½ for a direct transition semiconductor and 2 for an indirect transition semiconductor [38]. A and E_g_ represent the constant light frequency and bandgap energy, respectively. The bandgap energy was calculated from the plot of (αhν)^2^ versus hν plots. The bandgap energy of Mn_1_Fe_1_@SiO_2_, Mn_1_Fe_5_@SiO_2_, Mn_5_Fe_1_@SiO_2_, Mn_5_Fe_5_@SiO_2_, and Mn_20_Fe_20_@SiO_2_ was 1.62, 1.49, 1.78, 1.57, and 1.44 eV, respectively.

### 3.2. Dye Degradation Performance

#### 3.2.1. Effect of Metal Content on Performance 

**Figure 9:**
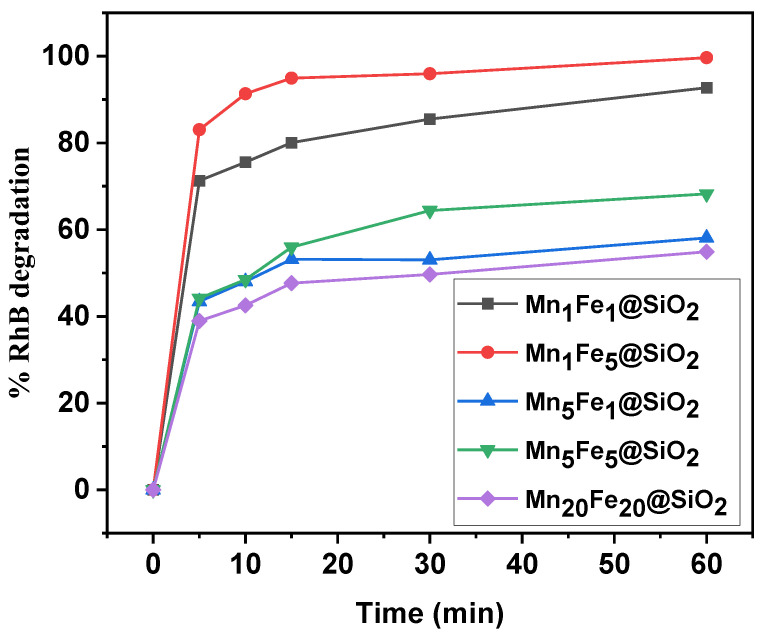
Comparison of synthesized nanocomposites for photocatalytic Fenton-like degradation of RhB.

All the synthesized nanocomposites were studied for their ability to degrade RhB dye under a photocatalytic Fenton-like process, and the results are shown in Figure 9. The graphs showed that Mn_1_Fe_5_@SiO_2_ had the highest removal performance (99.66%), and Mn_20_Fe_20_@SiO_2_ showed the lowest removal (54.89 %). These results can be attributed to the large surface area of Mn_1_Fe_5_@SiO_2,_ which can provide more active sites, increasing the removal efficiency. Based on these findings, experiments were continued using Mn_1_Fe_5_@SiO_2_, and the influencing parameters, including RhB initial concentration, catalyst dosage, H_2_O_2_ concentration, and UV intensity, have been investigated. 

#### 3.2.2. Effect of Experimental Parameters 

**Figure 10:**
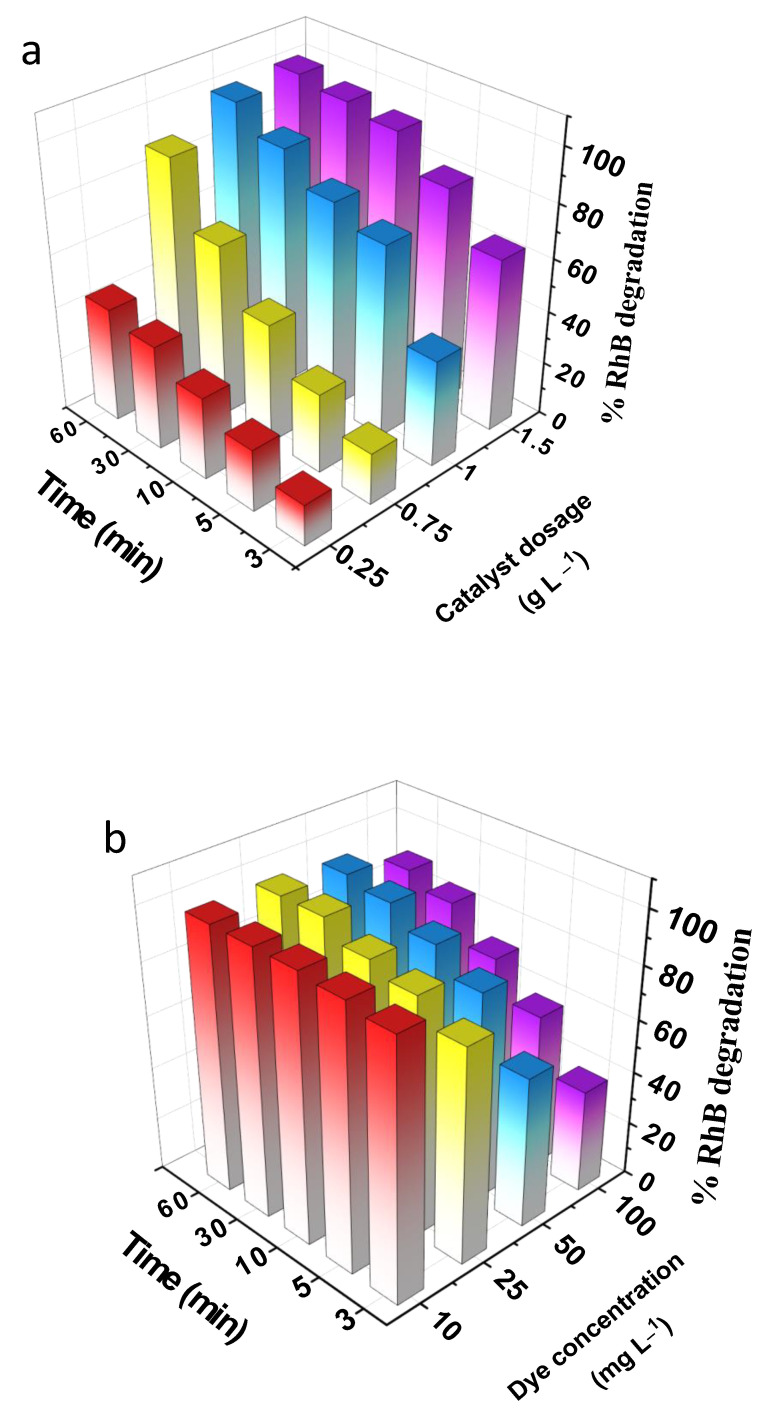

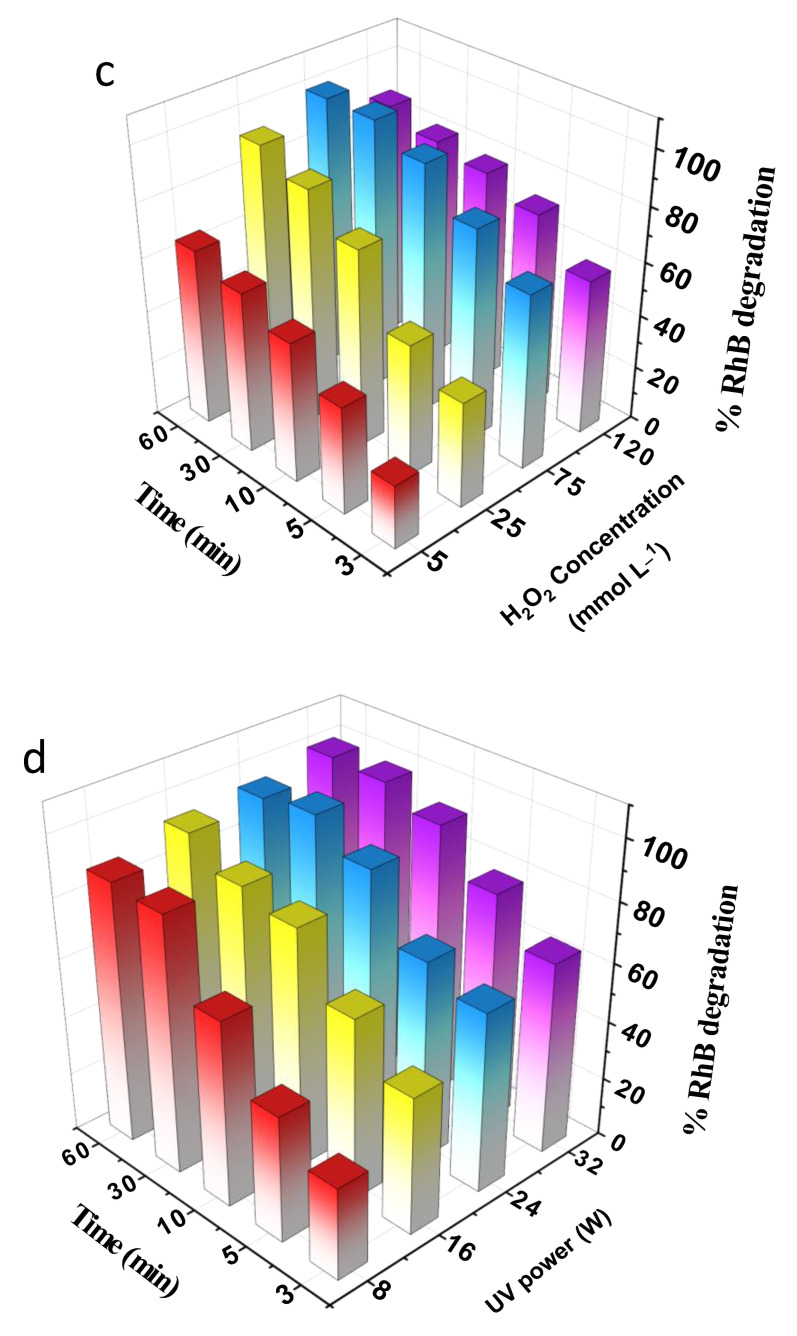
(**a**) Effect of catalyst dosage, (**b**) effect of initial concentration of RhB, (**c**) effect of H_2_O_2_ concentration, (**d**) effect of UV power on photocatalytic Fenton-like degradation of RhB using Mn_1_Fe_5_@SiO_2_.

**Catalyst dosage:** Figure 10a illustrates the effect of catalyst dosage on the degradation of RhB using Mn_1_Fe_5_@SiO_2_. In order to optimize the catalyst dosage, experiments were performed with different dosages of Mn_1_Fe_5_@SiO_2_ in the range of 0.25 to 1.5 g L^–1^. The removal efficiency was 43.2% and 99.8% for the 0.25 g L^–1^ and 1. 5 g L^–1^ catalysts, respectively. Increasing the dosage of Mn_1_Fe_5_@SiO_2_ can increase the presence of active sites and the hydroxyl radicals (^•^OH) formation, which finally enhances the removal performance. When the catalyst increased from 1 g L^–1^ to 1.5 g L^–1,^, the change in degradation efficacy was insignificant, and a dosage of 1 g L^–1^ was chosen to continue the experiments. 

**Initial concentration of RhB:** The effect of the initial concentration of RhB on removal efficiency of Mn_1_Fe_5_@SiO_2_ was evaluated with different RhB concentrations (10–100 mg L^–1^). Low dye concentrations (10 mg L^–1^ and 25 mg L^–1^) could be removed completely in the first 30 min. However, removal of high concentrations of 50 mg L^–1^ and 100 mg L^–1^ could reach 98.2 % and 90.6%, respectively, after the solution was irradiated for 60 min (Figure 10b). It can be seen that when the initial concentration of the RhB increased, it resulted in a decrease in degradation performance, which can be attributed to two main reasons. First, as the initial RhB concentration increases, more RhB molecules attach to the surface of the catalyst, decreasing the accessibility of active sites and minimizing the generation of e^−^/h^+^ pairs and hydroxyl radicals. Second, very high concentration of RhB inhibits UV/catalyst interactions because the dye can absorb the UV light and prevent it from being absorbed by the catalyst, which can also hinder the generation of electron/hole (e^−^/h^+^) pairs [39,40]. In this work, 50 mg L^−1^ of RhB was chosen as the optimized parameter.

**H_2_O_2_ concentration (electron acceptor):** The efficiency of the photocatalytic reaction increases by preventing electron/hole (e^−^/h^+^) recombination. Adding an electron acceptor such as H_2_O_2_ can effectively decrease the recombination of e^−^/h^+^. The degradation of RhB over Mn_1_Fe_5_@SiO_2_ was investigated using different concentrations of H_2_O_2_ (5 mmol L^−1–^120 mmol L^−1^) (Figure 10c). With an increase in H_2_O_2_ concentration from 5 mmol L^−1^ to 75 mmol L^−1^, the removal efficiency increased from 65.68% to 100%. Increasing the H_2_O_2_ concentration causes an increase in the removal performance because, under UV irradiation, the O–O bonds hydrogen peroxide could be broken to create hydroxyl radical (^•^OH). Therefore, more H_2_O_2_ concentrations can produce a higher number of (^•^OH) radicals. However, precautions are needed because, when H_2_O_2_ exceeds a certain level, it will act as a scavenger, decreasing dye degradation efficiency. In this study, by increasing the H_2_O_2_ concentration to 120 mmol L^−1^, the efficiency decreased to 88.2% [41,42]. Therefore, for subsequent experiments, 75 mmol L^−1^ of H_2_O_2_ was used.

**UV power:** The effect of UV power (8 W,16 W, 24 W, and 32 W) on RhB degradation was tested using Mn_1_Fe_5_@SiO_2_ (Figure 10d). Results showed that the removal was 88.21% for 8w UV power, which reached 100% as the UV power increased to 32 W. For higher UV powers, more photons can be formed and absorbed by the surface of the catalyst, which means more electron-hole (e^–^/h^+^) pairs can be generated. Consequently, the number of hydroxyl radicals accelerates and improves the removal performance. Moreover, in higher UV power, more hydroxyl radicals can be generated via H_2_O_2_ photodissociation, which means a greater number of ^•^OH radicals can be generated and attack the organic pollutants [43,44]. In this work, the power of 32 W was used as the optimized parameter. 

**Figure 11:**
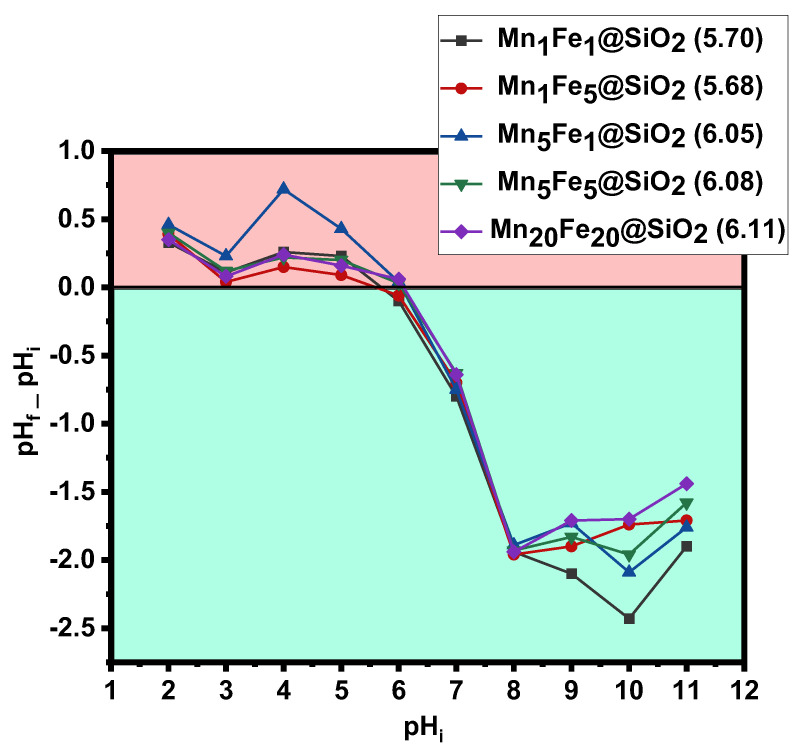
Zeta potential of used nanocomposites in this study.

**pHzpc**: The zero-point charge (pHzpc) value of photocatalysts was determined by pH drift method, as shown in Figure 11. The pHzpc value was found to be 5.7, 5.68, 6.05, 6.08, and 6.11 for Mn_1_Fe_1_@SiO_2_, Mn_1_Fe_5_@SiO_2,_ Mn_5_Fe_1_@SiO_2_, Mn_5_Fe_5_@SiO_2_, and Mn_20_Fe_20_@SiO_2_, respectively. The surface of nanocomposites was positively charged at pH below pHzpc, whereas it was negatively charged at pH above pHzpc. Since, in this experiment, the solution pH was 6.96 (neutral), the nanocomposite surface was negatively charged. 

#### 3.2.3. Degradation Performance in the Optimum Condition

**Figure 12:**
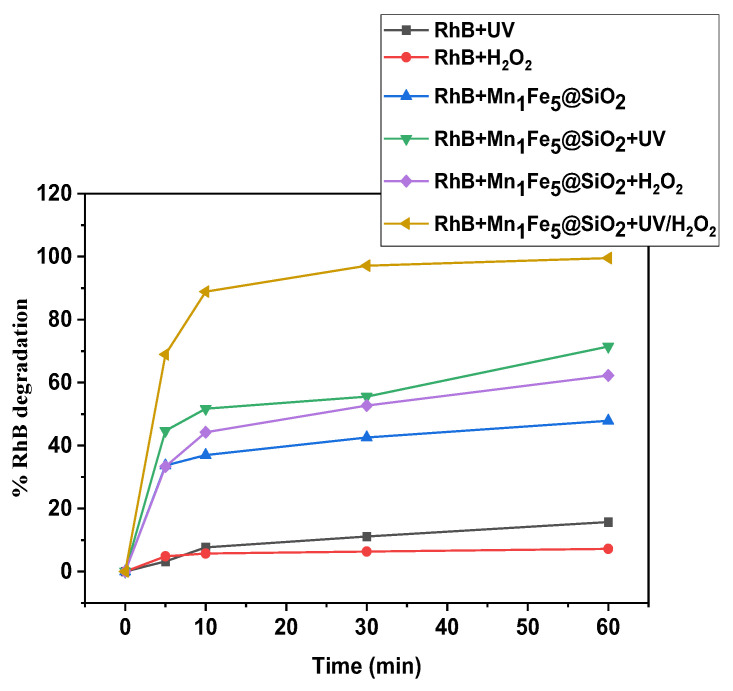
Degradation performance of Mn_1_Fe_5_@SiO_2_ in different systems in optimized condition [RhB] = 50 mg L^–1^, [Catalyst dosage] = 1 g L^–1^, [H_2_O_2_] = 75 mmol L^–1^, [UV intensity] = 32 W, [pH] = Neutral.

Mn_1_Fe_5_@SiO_2_ was studied for the degradation of RhB dyes in various removal methods. Figure 12 showed that, in the absence of the catalyst and using H_2_O_2_ or UV system, only 7.2∼15.7% of the dye could be degraded. Results indicated that 47.89% of RhB was adsorbed on Mn_1_Fe_5_@SiO_2_, indicating the catalyst capability in the adsorption, which is related to high surface area and more active sites. Moreover, based on Figure 11 pHzpc of Mn_1_Fe_5_@SiO_2_ is 5.68, and the pH of the RhB solution is 6.96; therefore, the surface charge of Mn_1_Fe_5_@SiO_2_ is negative in pH higher than pHzpc. Thus, there will be an attractive force between positive charge RhB and negative charge nanocomposite, which enhances the removal of RhB due to simultaneous adsorption /degradation at neutral pH. Complete removal of RhB was achieved under the heterogeneous photo-Fenton process. The results of this study have been compared with similar studies in Table 2. 

## 4. Conclusions

In this study, a series of novel metal oxide @silica nanocomposites (Mn_x_Fe_y_@SiO_2_) were synthesized for the first time via the sol-gel/combustion technique with different Mn and Fe contents (x,y = 1–20%). The results showed that the nanocomposite with the highest metal content (Mn_20_Fe_20_@SiO_2_) had better morphology (uniformed semi-spherical shape). Interestingly, BET results revealed a different behavior compared to the literature, and the lowest and highest surface area was expected to be M_20_Fe_20_@SiO_2_ and M_1_Fe_1_@SiO_2,_, respectively. However, in this study, among all the nanocomposites, the lowest surface area (*S*_BET_ = 320.74 m^2^/g) belongs to M_5_Fe_1_@SiO_2,_ which has a high Mn and low Fe content, and the highest specific surface area of 654.95 m^2^/g belongs to the sample with more Fe and less Mn content (M_1_Fe_5_@SiO_2_). From these results, it could be concluded that increasing Mn has a more substantial effect compared to Fe in decreasing the surface area. Moreover, the ratio of Mn and Fe appears to have a more tangible effect on surface characteristics compared to the effectiveness of Fe and Mn content. UV–Visible DRS data showed that all the nanocomposites have strong absorbance in the entire UV–Visible range with bandgap energy of 1.44–1.78 eV. XRD and ESR results indicated that Mn existed in a mixture valence of Mn(III) (Mn_2_O_3_) and Mn(II) (Mn_3_O_4_) in all the nanocomposites. 

The application of these nanocompo”Ite’ was demonstrated by the decolorization of Rhodamine B (RhB) under a photocatalytic Fenton-like reaction. In the optimized experimental conditions, the Mn_1_Fe_5_@SiO_2_ showed the highest dye removal due to a higher number of active sites, which could increase dye degradation. Additionally, electrostatic attraction between the surface of the nanocomposite with a negative charge and positive charge RhB made the nanocomposite dual functional material where the adsorption/degradation could occur, simultaneously enhancing the removal performance. Thus, this study recommends a promising method for fabricating an efficient nanomaterial with potential applications to remove complex organic pollutants at neutral pH.

## Figures and Tables

**Table 1:** The synthesized nanocomposites.

Catalyst	Content	Color of Formed Gel
Mn_1_Fe_1_@SiO_2_	Lowest metal content (Mn:1%, Fe:1%)	Yellow
Mn_1_Fe_5_@SiO_2_	Higher Fe content (Mn:1%, Fe:5%)	Light brown
Mn_5_Fe_1_@SiO_2_	Higher Mn content (Mn:5%, Fe:1%)	Yellow
Mn_5_Fe_5_@SiO_2_	Medium metal content (Mn:5%, Fe:5%)	Light brown
Mn_20_Fe_20_@SiO_2_	Highest metal content (Mn:20%, Fe:20%)	Dark brown

**Table 2:** Comparison of the performance of the synthesized nanomaterial with other studies.

Adsorbent/Catalyst	Pollutant	Experimental Condition	Removal	References
TiO_2_	[ATL]_0_ 10 mg L^−1^	pH3, Temperature = 25 °C, [catalyst] = 250 mg L^−1^, [H_2_O_2_] = 1.4 Mm, Reactiontime = 2 h, UVA9W (350 nm)	70%	[45]
SBA-15	[IBU]_0_ 25 ug L^−1^	pH5, Temperature=25 °C, [catalyst] = 0.3g L^−1^, [H_2_O_2_] = 10 Mm, Reaction time = 12 h	93%	[46]
Mn_2_O_3_	[MB]_0_ 15 mg L^−1^	pH not mentioned, Temperature = 25 °C, [catalyst] = 1g L^−1^, [H_2_O_2_] = 2 Ml (%30), Reaction time = 3 h	50%	[47]
G0-TiO_2_	Methyl orange 20 mg L^−1^	pH6.2, Temperature = 25 °C, [catalyst] = 0.5g L^−1^, Reaction time = 2 h, UVC	53%	[48]
Mn_1_Fe_5_@SiO_2_	[RhB]_0_ 50 mg L^−1^	pH7,Temperature = 25 °C, [catalyst] = 1 g L^–1^, [H_2_O_2_] = 75 mmol L^–1^, Reactiontime = 1 h, UVC-32 W (254 nm)	100%	This study

## Supplementary Materials

The following supporting information can be downloaded at: https://www.mdpi.com/article/10.3390/nano12234108/s1, Figure 1: SEM image of samples (a) Mn_1_Fe_1_@SiO_2_, (b) Mn_1_Fe_5_@SiO_2_, (c) Mn_5_Fe_1_@SiO_2_, (d) Mn_5_Fe_5_@SiO_2_, (e) Mn_20_Fe_20_@SiO_2_; Figure 2: HRTEM image of samples (a) Mn_1_Fe_1_@SiO_2_, (b) Mn_1_Fe_5_@SiO_2_, (c) Mn_5_Fe_1_@SiO_2_, (d) Mn_5_Fe_5_@SiO_2_, (e) Mn_20_Fe_20_@SiO_2_.
