# Supplementary material for: A Novel Metal-Containing Mesoporous Silica Composite for the Decolorization of Rhodamine B: Effect of Metal Content on Structure and Performance
Source: Nanomaterials (Basel). 2022 Nov 22;12(23):4108. doi: 10.3390/nano12234108 (PMC9736502; doi:10.3390/nano12234108)
Supplement: Supplementary file 1 [file nanomaterials-12-04108-s001.zip › nanomaterials-1997061-supplementary.pdf]

Supplementary Materials

A Novel Metal-Containing Mesoporous Silica Composite for the Decolorization of Rhodamine B: Effect of Metal Content on Structure and Performance

Yasaman Ghaffari [1,2], Md Saifuddin [3], Suho Kim [4], Soyoung Beak [2], Jiyeol Bae [1,2] and Kwang Soo Kim [1,2,*]

1 Department of Environmental Research, Korea Institute of Civil Engineering and Building Technology (KICT), University of Science and Technology (UST), Daejeon 34113, Republic of Korea
2 Department of Environmental Research, Korea Institute of Civil Engineering and Building Technology (KICT), Goyang 10223, Republic of Korea
3 Civil and Environmental Engineering Department, Hanyang University, Seoul 04763, Republic of Korea
4 Department of Civil & Environmental Engineering, Yonsei University, Seoul 03722, Republic of Korea
* Correspondence: kskim@kict.re.kr

The surface morphology of photocatalysts was analyzed by field emission scanning electron microscopy (FE-SEM) (Hitachi S-4300, Tokyo ,Japan). The dried samples were coated with a gold-platinum alloy by ion-sputtering (E-1048 Hitachi ion sputter). The transmission electron microscope (TEM) images were recorded with a field emission TEM (FE-TEM, JEM-2010F, JEOL, Tokyo, Japan). The two-dimensional (2D) elemental mapping was done by energy-dispersive X-ray spectroscopy (EDS) (X-Maxn 80 T, Oxford, England, UK). Thermal gravimetric analysis (TGA) were investigated using Thermogravimetric Analyzer (TG 209 F3, NETZSCH Selb, Germany). For Brunauer-Emmett-Teller (BET) analysis, N_2 adsorption-desorption isotherm was measured at 77 K in a Gemini series Micromeritics 2360 instrument. Before subjecting for BET analysis, samples were degassed at 473 K for 2 h with a Micromeritics FlowPrep 060. The diffraction patterns were obtained using Ultima IV(Rigajku, Tokyo, Japan) X-ray diffractometer (XRD) with Cu Kα and a Ni filter where the scanning speed was set to 3° min^{-1}. Fourier-transform

infrared (FTIR) spectra of samples were recorded using KBr pellets over a FTIR spectrometer (Cary670, USA, California, Agilent). The recording was done with a single-beam spectrometer with 60 added scans. Ultraviolet-visible light-diffuse reflectance spectroscopy (UV-Vis DRS) spectra of photocatalysts were obtained using a SCINCO S-4100 spectrometer equipped with a photodiode array detector and a diffuse reflectance attachment. Before analysis, all the samples were dehydrated in the air at 673 K for 7 h. For X-ray photoelectron spectroscopic (XPS) analysis of samples, a K-alpha XPS instrument (Thermo Scientific Inc., East Grinstead, England UK) with a monochromatic Al Kα X-ray source was used where the pressure was fixed to 4.8×10^{-9} mbar. The electron spin resonance (ESR) measurement was carried out by a JOEL JES-FE1C X-band spectrometer(Tokyo, Japan).

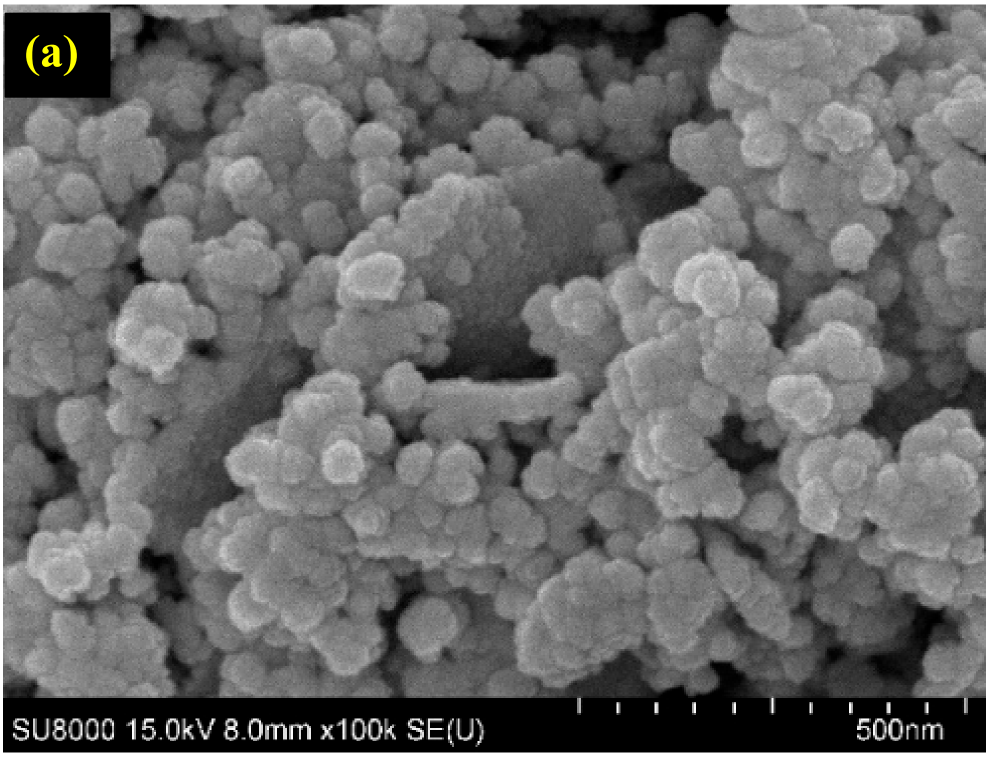

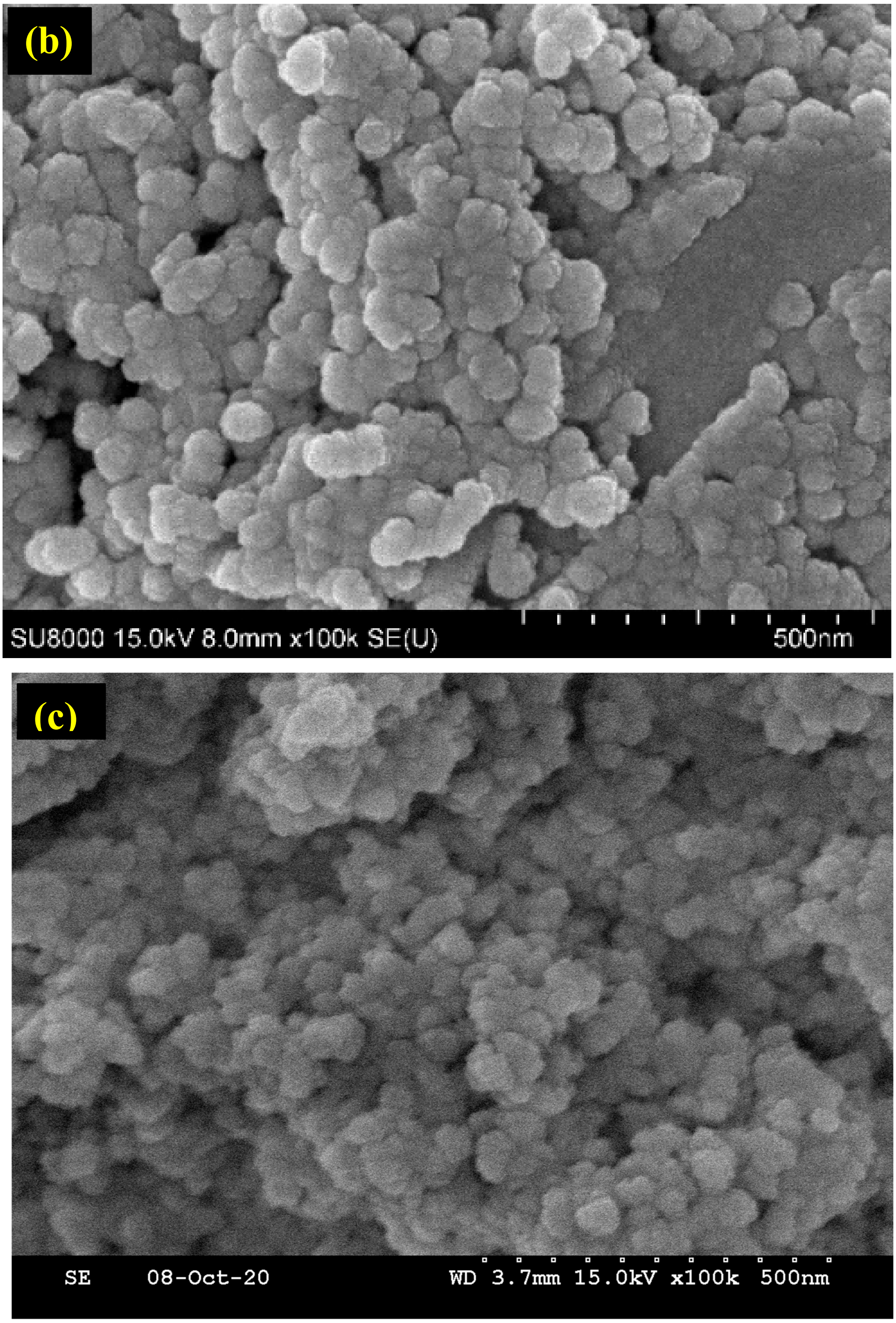
(b)
SU8000 15.0kV 8.0mm x100k SE(U)
500nm
(c)
SE
08-Oct-20
WD 3.7mm 15.0kV x100k 500nm

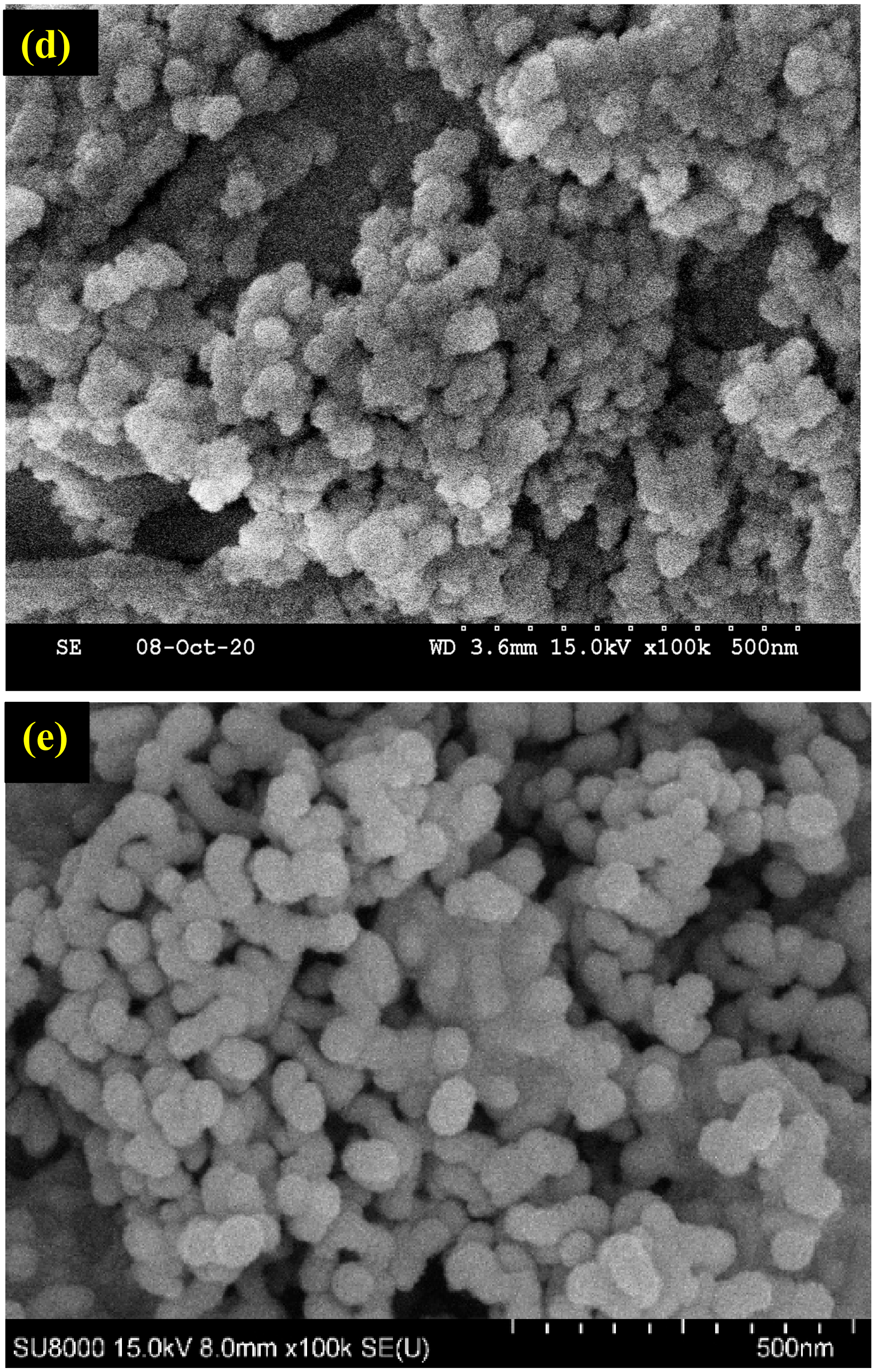

Figure S1. SEM image of samples (a)Mn_1Fe_1@SiO_2, (b)Mn_1Fe_5@SiO_2(c), Mn_5Fe_1@SiO_2(d), Mn_5Fe_5@SiO_2, (e) $Mn_{20}Fe_{20}$@SiO_2.

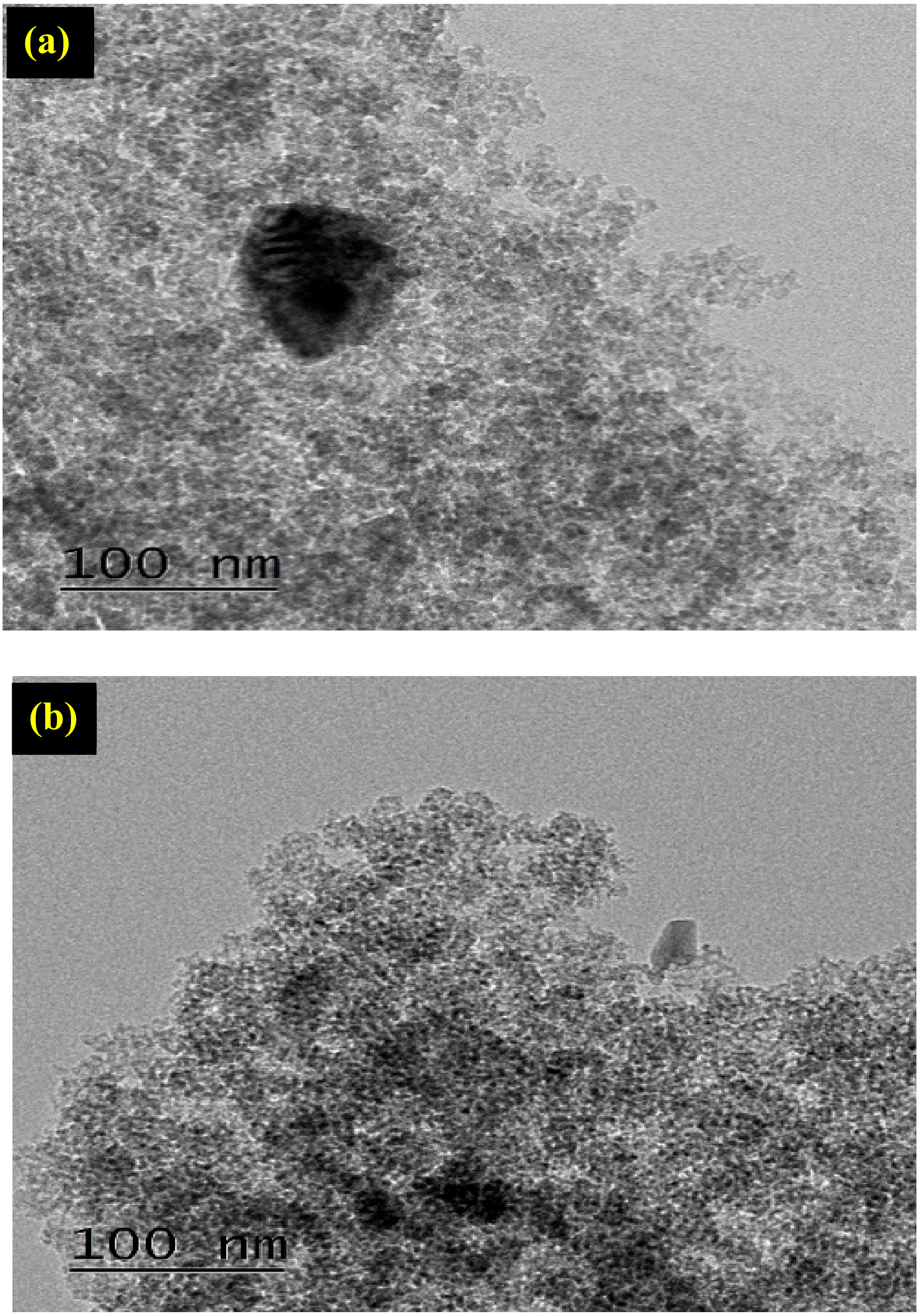
(a)
100 nm
(b)
100 nm

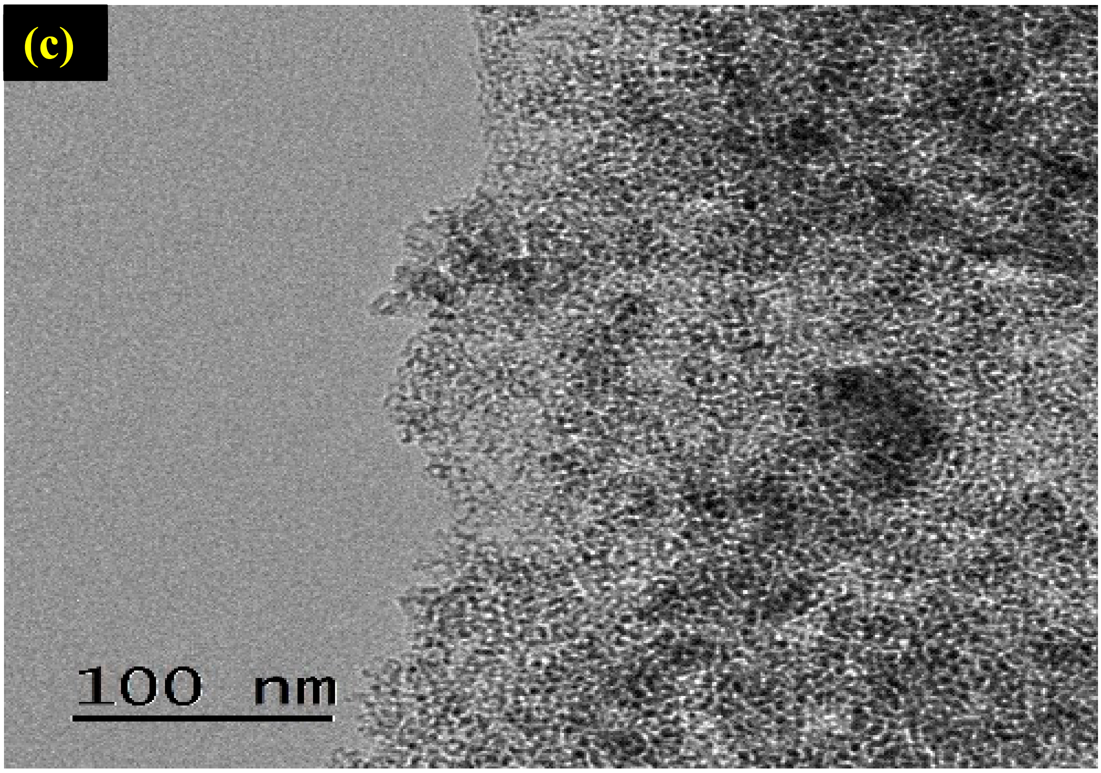
(c)
100 nm

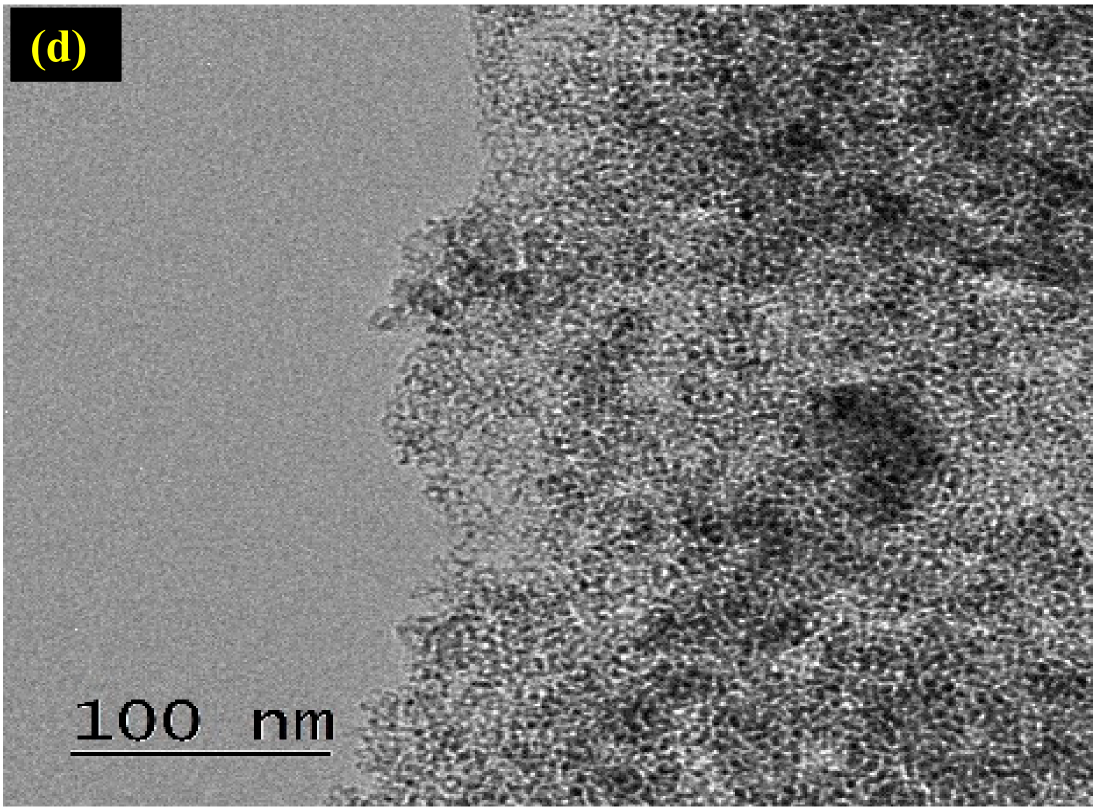
(d)
100 nm

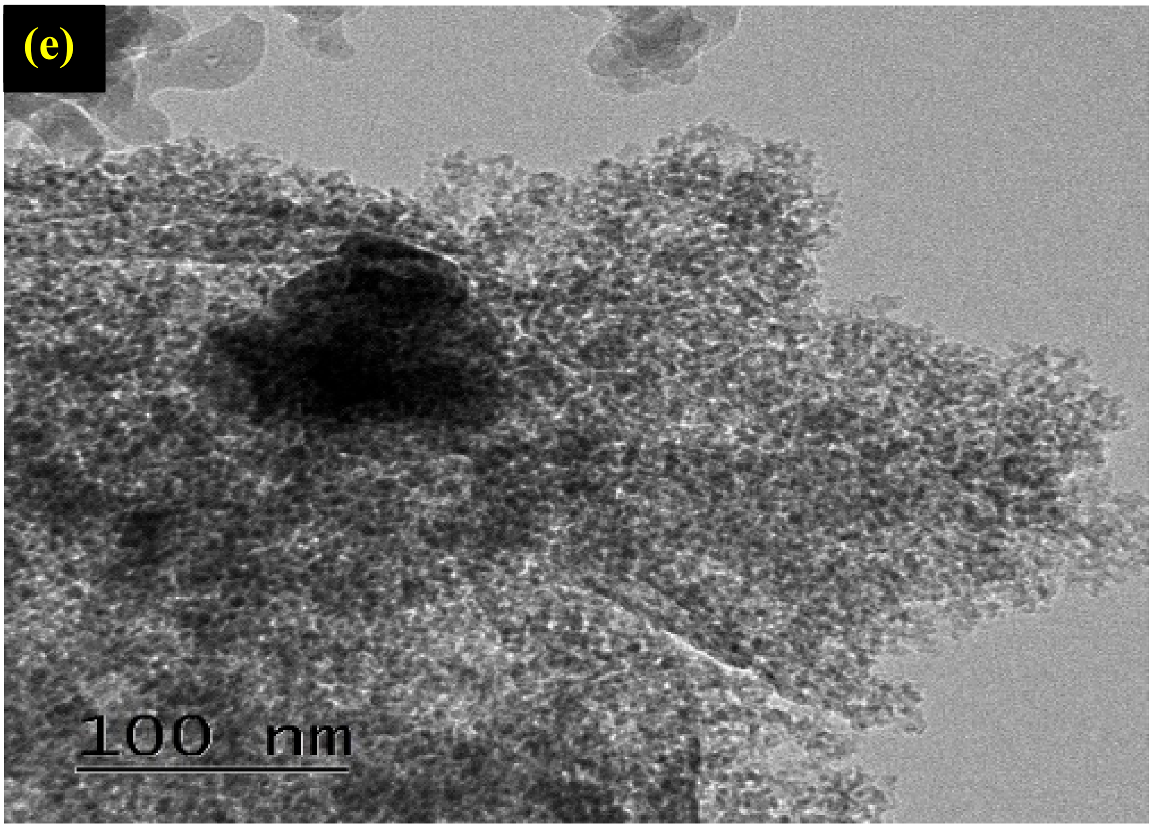

Figure S2. HRTEM image of samples (a)$Mn_1Fe_1@SiO_2$, (b)$Mn_1Fe_5@SiO_2$(c), $Mn_5Fe_1@SiO_2$(d), $Mn_5Fe_5@SiO_2$, (e) $Mn_{20}Fe_{20}@SiO_2$.
